# 

**Published:** 1881-04

**Authors:** 


### CONTENTS FOR APRIL, 1881.

ORIGINAL COMMUNICATIONS.

ART. I.—Treatment of Mammary Abscess. By HIRAM CORSON, M. D. .......... 151
ART. II.—Anæsthetics and Narcotics. By LAURENCE TURNBULL, M. D. ........ 161
ART. III.—Adam a Nation. By W. H. RUFF, M. D. 171
Translation .......... 174

EDITORIAL DEPARTMENT.

Hypnoptism, Mesmorism, or Syggnosticism.... 175
Much Craniotomy. Bellevue Medical College.. 176
A City Hospital.......... 177
Decimal Arrangement.......... 178
Night Suit for Physicians.......... 179
Heart Troubles & Cactus Grandiflora & Cimicifuga 179
Colorado and its Climate. Fraternal Journalism. 180
Popular Science.......... 181
Prof. Remsen's Report on Baltimore Water.... 181
Juglans Nigra, Black Walnut in Diphtheria.... 181
Quinine in Whooping Cough.......... 182
The Difference in the Count.......... 182
New Medical College in Philadelphia.......... 183
Death of Prof. Richard O. Cowling, A. M., M. D. 183
Annual Meeting of the State Medical Society... 183
Buffalo Lithia Water.......... 184
The North American Review. Dental Fees... 184
Meeting of Dental Societies in May.......... 222

BIBLIOGRAPHICAL DEPARTMENT.

The Scientific American and Supplement...... 186
A Practical Treatise of the Skin.......... 186
Imperfect Hearing and the Hygiene of the Ear.. 187
Strangulated Veins of the Uterus.......... 188
Unias Medica. Medical Times and Gazette.... 188
The Homœopathic Times.......... 188
Johns Hopkins University Circular.......... 188
Pacific Medical and Surgical Journal.......... 188
The Detroit Lancet.......... 188
North American Review. The Pathologist.... 189
Sanitary Engineer.......... 190
First Biennial Report of the North Carolina Board of Health.......... 190
Dr. I. W. Singleton.......... 190
El Medico Y Cirujano Centro-Americano...... 190
Kansas City Review of Science and Industry... 190

THERAPEUTIC PEARLS AT RANDOM STRUNG....190–194
EXCERPTA ..........195–202

POPULAR SCIENCE DEPARTMENT.

Foot Prints in the Rock.......... 203
Microscopical Analysis of Water.......... 205
Cement for Rubber.......... 208
The Photophone—Transmission of Sound by Light.......... 209
Causes of Heat in Mines.......... 210
Rate of Growth of Coral.......... 212
Black Races of Oceanica.......... 212
Out-Door Air and Exercise.......... 213
American Arctic Research.......... 214
Prehistoric Mining in North Carolina.......... 216
Rock-Boring Ephemera.......... 217
M. Faye's Theory of the Crust of the Earth..... 217
Only a Vine-Slip.......... 218
Telegraphing without Wires.......... 221
NECROLOGICAL .......... 222
Mortuary Report .......... 224

